# Evaluation of the vaginal and urinary microbiota of healthy cycling bitches

**DOI:** 10.1186/s12917-024-04104-w

**Published:** 2024-07-15

**Authors:** Virginie Gronsfeld, Flore Brutinel, Sophie Egyptien, Charles Porsmoguer, Annick Hamaide, Bernard Taminiau, Georges Daube, Marie-Lys Van de Weerdt, Stefan Deleuze, Stéphanie Noel

**Affiliations:** 1 Department of Companion Animal Clinical Sciences, Faculty of Veterinary Medicine, University of Liège, Liège, Belgium; 2 Department of Food Sciences, Faculty of Veterinary Medicine, University of Liège, Liège, Belgium; 3 Labforvet, Rue Tienne aux Grives 18, Andenne, 5300 Belgium

**Keywords:** Vaginal microbiota, Urinary microbiota, Hormonal influence, Dogs, Estrous cycle

## Abstract

**Background:**

While the urogenital microbiota has recently been characterized in healthy male and female dogs, the influence of sex hormones on the urogenital microbiome of bitches is still unknown. A deeper understanding of the cyclic changes in urinary and vaginal microbiota would allow us to compare the bacterial populations in healthy dogs and assess the impact of the microbiome on various urogenital diseases. Therefore, the aim of this study was to characterize and compare the urogenital microbiota during different phases of the estrous cycle in healthy female dogs. DNA extraction, 16 S rDNA library preparation, sequencing and informatic analysis were performed to determine the vaginal and urinary microbiota in 10 healthy beagle dogs at each phase of the estrous cycle.

**Results:**

There were no significant differences in alpha and beta diversity of the urinary microbiota across the different cycle phases. Similarly, alpha diversity, richness and evenness of vaginal bacterial populations were not significantly different across the cycle phases. However, there were significant differences in vaginal beta diversity between the different cycle phases, except for between anestrus and diestrus.

**Conclusion:**

This study strongly suggests that estrogen influences the abundance of the vaginal microbiota in healthy female dogs, but does not appear to affect the urinary microbiome. Furthermore, our data facilitate a deeper understanding of the native urinary and vaginal microbiota in healthy female dogs.

**Supplementary Information:**

The online version contains supplementary material available at 10.1186/s12917-024-04104-w.

## Background

In recent years, there has been a growing interest in the role of the microbiome in health and disease. Early studies of the urogenital microbiome primarily relied on culture-based techniques. Unfortunately, these techniques often failed to detect most of the resident microflora [1]. In human medicine, more recent studies have used 16 S rDNA gene sequencing to show that urine is not sterile [2]. In dogs, studies established that samples collected via cystocentesis differ from those collected via midstream voiding [3]. Genome phylogenetic analysis of bacterial strains isolated from the vagina and bladder of women suggest that the female urogenital microbiota is interconnected, comprising various health-associated commensals, such as *Lactobacillus, Corynebacterium, Streptococcus, Actinomyces, Gardnerella* and *Bifidobacterium* species [2–10]. Furthermore, sex hormones contribute to the regulation of the vaginal microbiota in women, which may modify mucosal estrogen levels [11–13]. However, while the vaginal microbiota varies between prepubertal, pubertal and post-menopausal women [14, 15], in most women, it remains relatively stable throughout the menstrual cycle, with little variation in diversity and only modest fluctuations in species richness [16].

**Fig. 1:**
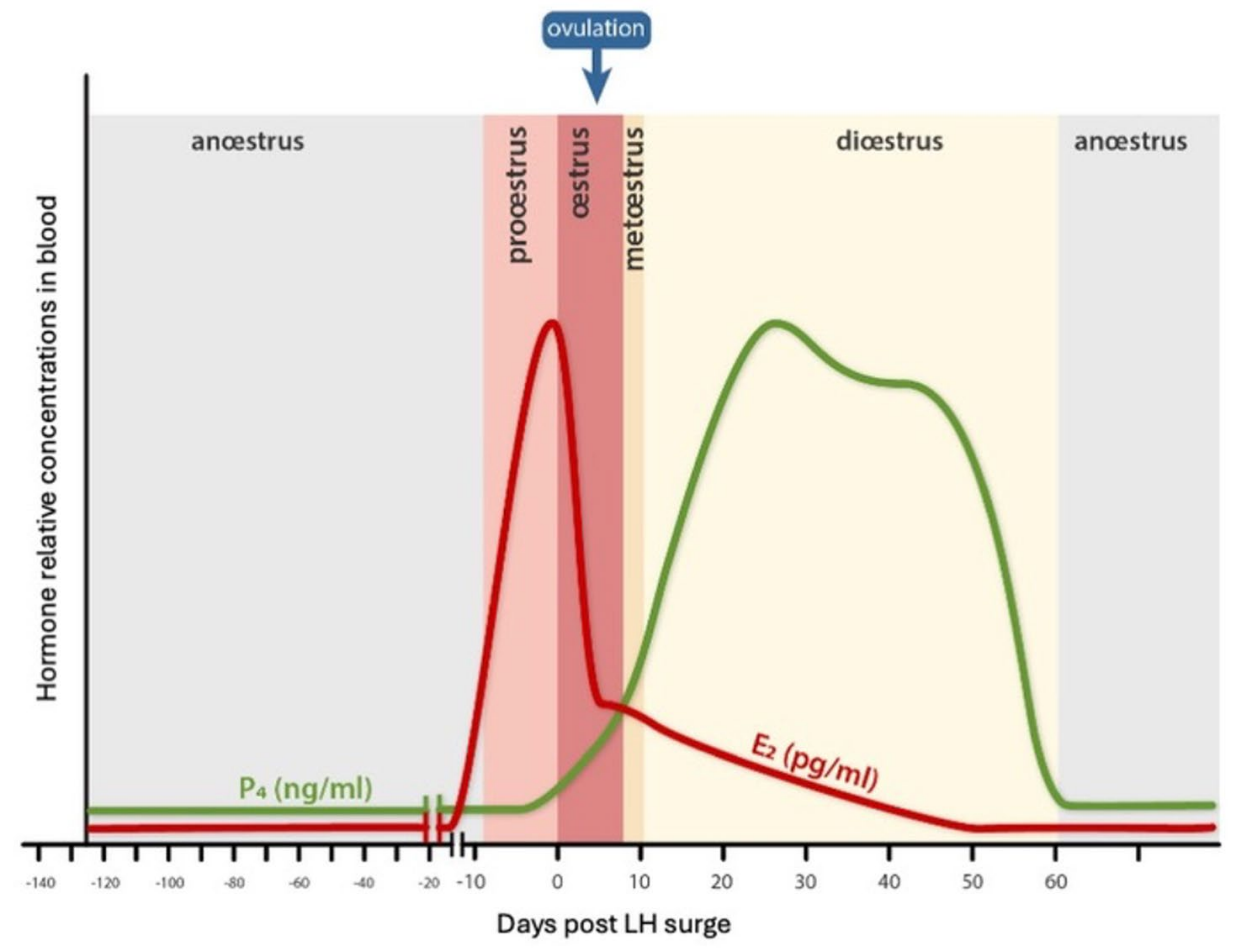
Estrogen and Progesterone modifications during female dog cycle

In veterinary medicine, 16 S rDNA gene sequencing has recently been used to characterize the urogenital microbiome in healthy dogs. Four taxa, belonging to the *Pseudomonadota* (previously *Proteobacteria)* phylum: *Pseudomonas* spp, *Acinetobacter* spp, *Sphingobium* spp and *Bradyrhizobiaceae*, dominated the urinary microbiota in dogs of both sexes. Moreover, considerable overlap was observed between the vaginal and bladder microbiota where *Pseudomonas* and *Acinetobacter* were the most abundant taxa [6]. Another recent study showed that *Hydrotalea*, *Ralstonia*, *Mycoplasma*, *Fusobacterium* and *Streptoccocus* were the predominant species in the vagina of female dogs [17]. The vaginal microbiota of bitches was most diverse, with the highest richness, during the estrous phase of the estrous cycle. However, these differences were only statistically significant between estrous and the prepubertal stage [17]. These results may be related to the age of the dogs in the study as the diversity of the vaginal microbiome continuously changes with age [18]. For the good understanding of the paper, the main hormonal changes and phases of the cycle are illustrated in Fig. 1.

The urinary system is also affected by sex hormones and the estrous cycle [19]. Urodynamic studies in dogs have shown that urethral pressure, an indicator of urinary continence, decreased during estrous and early diestrus [20]. However, the influence of sex hormones on the urinary microbiota of female dogs is still unknown. Only, difference in urinary microbiota between pre- and post-menopausal women was demonstrated [21, 22].

Therefore, the purpose of our research was to characterize and compare the urogenital microbiota in the different phases of the estrous cycle in healthy bitches. Since the bladder and the vaginal microbiota are closely connected, we hypothesized that sex hormones and the estrous cycle influence both microbiotas.

## Methods

### Study population

This prospective study was conducted on 10 healthy intact adult female laboratory beagle dogs owned by the Veterinary Faculty of the University of Liège (ULiège ethical approval number: 20-2250; laboratory approval number: LA161012). Dogs were included in the study if they had no signs of systemic, vaginal or lower urinary tract disease. Dogs that had received antibiotics, probiotics and/or anti-inflammatory drugs within 30 days prior to enrolment were excluded. Dogs were housed in a kennel with wood shavings as bedding.

### Study design

Samples were obtained from adult dogs at the following phases of the estrous cycle: proestrus, estrous, diestrus and anestrus. Phases of the estrous cycle were identified via visual examination of the vulva, cytologic examination of a vaginal smear and measurement of plasma progesterone concentration (Automated Immunoassay analyzer 360, TOSOH). Urine was collected from all dogs via prepubic cystocentesis after skin disinfection with chlorhexidine soap and alcohol to minimize dermal microbiota contamination. Around 10 ml of urine was aliquoted for routine analysis (specific gravity, dipstick with pH, and microscopic evaluation of the sediment), routine culture and 16 S rRNA amplicon sequencing at each cycle phase. The culture has been performed by mass spectrophotometry (VITEK MS biomérieux) and conventional biochemistry (VITEK 2 Biomérieux). After placing a sterile (UV treatment for 30 min in a BSL2 Biohazard cabinet) otoscope cone beyond the vestibule, a swab moistened with sterile saline solution was passed through the speculum into the anterior vagina that was swabbed for 10 s to collect a genital sample. Negative controls consisted of saline moistened vaginal swabs passed through a sterile speculum. Urine and vaginal samples were stored at -80 °C until DNA extraction.

### Total bacterial DNA extraction and amplicon sequencing

16 S rDNA library preparation, sequencing and informatic analysis were performed as previously described [23]. Briefly, total bacterial DNA were extracted from the vaginal swabs and urine with the DNEasy Blood and Tissue kit (QIAGEN Benelux BV; Antwerp, Belgium) with an added bead beating step during lysis. Amplicon sequencing targeting V1V3 hypervariable regions of the 16 S rDNA was performed using a MiSeq sequencer (Illumina; San Diego, California, USA). Sequence reads were cleaned and processed using MOTHUR software package v1.47 and the SILVA v1.38_1 16 S rDNA reference database. The urinary and vaginal microbiota were analyzed separately following the same protocol. Analyses were performed at the genus taxonomic level.

The identification of putative bacterial contaminant population in vaginal swab involves the molecular quantification of the bacterial load in samples. This protocol is described elsewhere and is based upon the quantitative amplification of the V2V3 hypervariable region of the 16 S rDNA by real time PCR [24].

### Statistical analysis

Good’s coverage index and ecological indicators, including the α-diversity (inverse Simpson’s index and Shannon index), bacterial richness (Chao1 index) and evenness (Simpson index-based measure) were calculated with MOTHUR v1.47. Differences between groups were assessed using non parametric Friedman ANOVA test for repeated measures, followed by paired post-hoc tests corrected with a two-stage linear step-up Benjamini Hochberg procedure (q threshold = 0.05) with PRISM 9.0.

Beta diversity was visualized with a Bray–Curtis dissimilarity matrix-based non-parametric dimensional scaling (NMDS) model using vegan and vegan3d packages in R. Differences in beta diversity between cycle phases was assessed with analysis of molecular variance (AMOVA) and homogeneity of molecular variance (HOMOVA) tests using MOTHUR (using 10,000 iterations on the subsampled table) on a Bray–Curtis dissimilarity matrix. A p-value < 0.05 was considered significant.

Differential population abundance between cycle phases was evaluated using a negative binomial Wald test in the DESeq2 R package. Differences were considered significant if the corrected p-value was < 0.05 (Benjamini-Hochberg False Discovery Rate multi-testing correction).

To identify putative bacterial contaminants in vaginal swabs sample, a multiple non-parametric Spearman Rho correlation test was performed between genus abundance and total bacteria load, determined by real time PCR. The correlation was considered as significant for Rho value above 0.5 or below − 0.5, with a p-value < 0.05. We also use Decontam package [25] in R to detect putative contaminants in the bacterial profiles, following authors protocols for the prevalence and frequency strategies. For the first strategy, sample library DNA quantification was used. For the second strategy, specific negative controls for vaginal swabs and sequencing run negative controls for the urine samples were used.

A Matrix correlation Mantel test [26] was performed with the Pearson, Spearman and Kendall test to evaluate the correlation between vaginal and urinary microbiota.

## Results

### Study population characteristics

The mean weight of the dogs included in this study was 14.25 kg (range 12–17 kg). The mean age was 6.5 years (range 5–9 years). Dogs were housed together in a kennel with wood shavings as bedding. They were fed with a strict diet of adult maintenance or light dry food.

### Urine analysis

Urine pH was between 4.5 and 8 and urine specific gravity was between 1.005 and 1.048. Mild proteinuria was frequently observed in five dogs and consistently observed in two dogs (Table 1).

### Urine bacterial cultures

Urine cultures were mostly negative, with the exception of *Streptococcus infantarius* and *Escherichia coli* in one estrous sample, *Enterococcus hirae* in the diestrus sample from one dog and *Lactobacillus gasse* in the estrous sample from one dog.

**Table 1:** Urinalysis and urinary culture

Beagle	Phase	Dipstick	Specific gravity	Sediment	Urinary culture
1	Proestrus	pH 7	1,016	negative	negative
2		pH 7, proteins +	1,005	negative	negative
3		pH 6	1,014	negative	
4		pH5	1,018	negative	negative
5		pH 7	1, 024	negative	negative
6		pH5, proteins +	1,04	negative	negative
7		pH 8	1,015	negative	negative
8		pH 6,5, blood +, bilirubin +	1,024	negative	negative
9		pH5, blood ++, bilirubine +, proteins +	1,024	Epithelial cells and Erythrocyts	negative
10		pH 6, proteins +	1,03	Epithelial cells	negative
1	Estrous	pH 6	1,016	negative	negative
2		pH 7, proteins++	1,027	negative	80% Streptococcus infantarius, 20% E. Coli
3		pH 7, blood +	1,024	Epithelial cells and extracellular bacteria	100% Lactobacillus gasseri
4		pH 5	1, 022	negative	negative
5		pH 7	1,014	Epithelial cells	negative
6		pH5 +, proteins +	1,027	negative	negative
7		pH 5	1,024	negative	negative
8		pH 8	1,008	negative	negative
9		pH 5, blood +, proteins ++	1,02	Erythrocyts	negative
10		pH 7 proteins +	1,032	negative	negative
1	Diestrus	pH7, blood+, bilirubin +, proteins +	1,026	negative	negative
2		pH 6, bilirubin +, proteins +	1,027	negative	100% Enterococcus hirae
3		pH 5	1,028	negative	negative
4		pH 5	1, 022	negative	negative
5		pH 8	1,012	negative	negative
6		-	-	-	negative
7		pH 6,5, proteins +	1, 024	negative	negative
8		-	-	-	negative
9		pH6, proteins +	1,014	negative	negative
10		pH 6	1,034	Epithelial cells	negative
1	Anestrus	pH 7	1,018	negative	negative
2		pH 8, proteins ++	1,01	negative	negative
3		pH 6, proteins+	1,032	negative	negative
4		pH 5, proteins ++	1, 022	negative	negative
5		pH 7	1,017	negative	negative
6		pH 4,5, proteins +	1, 020	negative	negative
7		pH 6,5, proteins +	1,028	Erythrocyts	negative
8		pH 5	1,02	negative	negative
9		pH 5	1,02	negative	negative
10		pH 6,5 Proteins +	1,048	negative	negative

### Vaginal microbiota

**Fig. 2:**
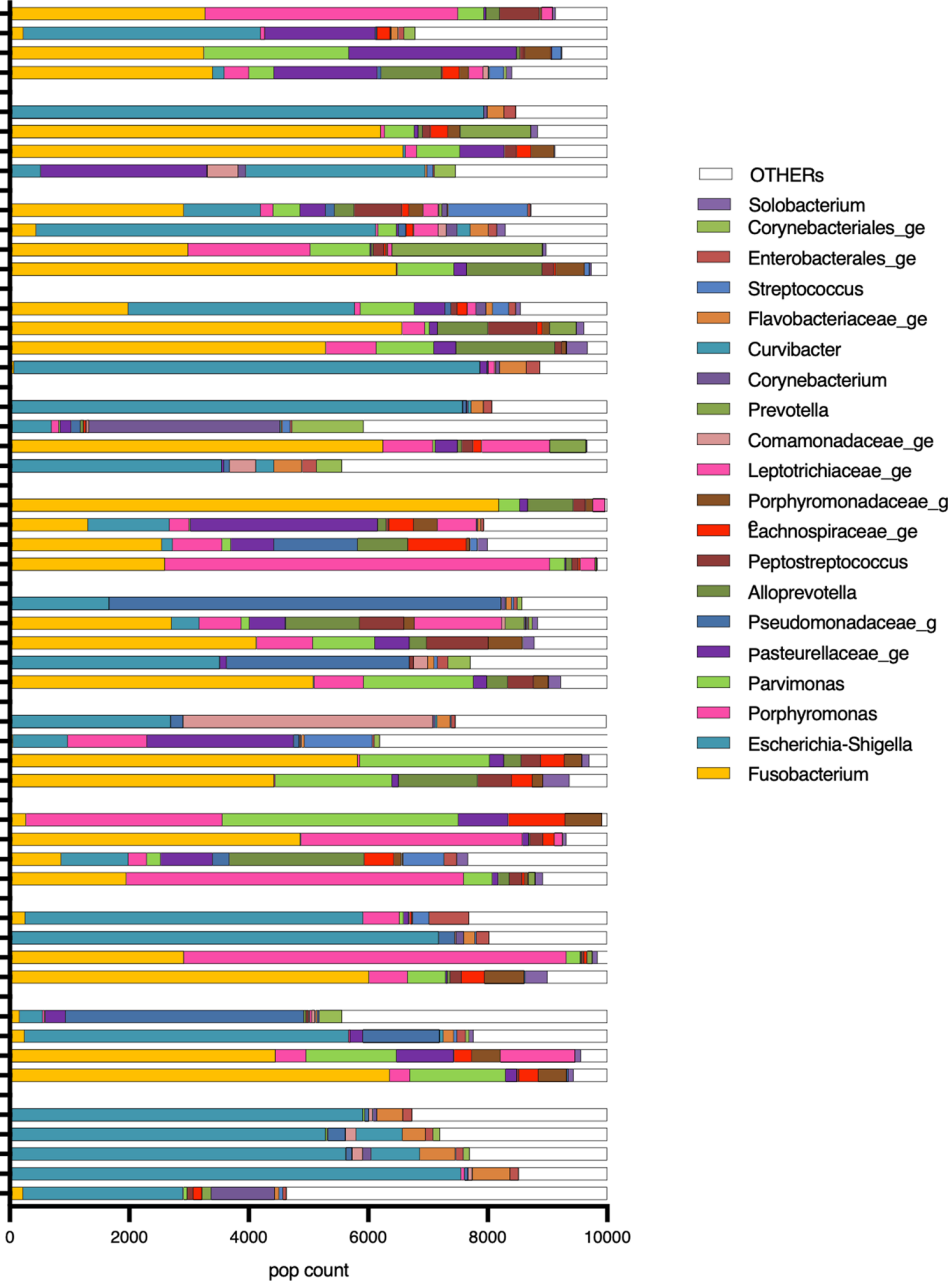
Vaginal genus

A total of 315 genera were identified in the vaginal samples during the different phases of the estrous cycle. The most abundant bacterial populations belonged to the genera *Fusobacterium*, *Porphyromas*, *Parvimonas* and *Escherichia-Shigella*, which represented 33.1%, 11.5%, 7.1%, 7% and 5.8%, respectively, of the total bacterial population (Fig. 2). To investigate whether the identified bacterial genera were contaminants, a non-parametric Spearman correlation test was performed between the abundance of each identified genus and the total bacterial population. The negative result (Rho − 0,7972; *p* < 0.0001) for *Escherichia-Shigella* suggested that it was a contaminant.

**Fig. 3:**
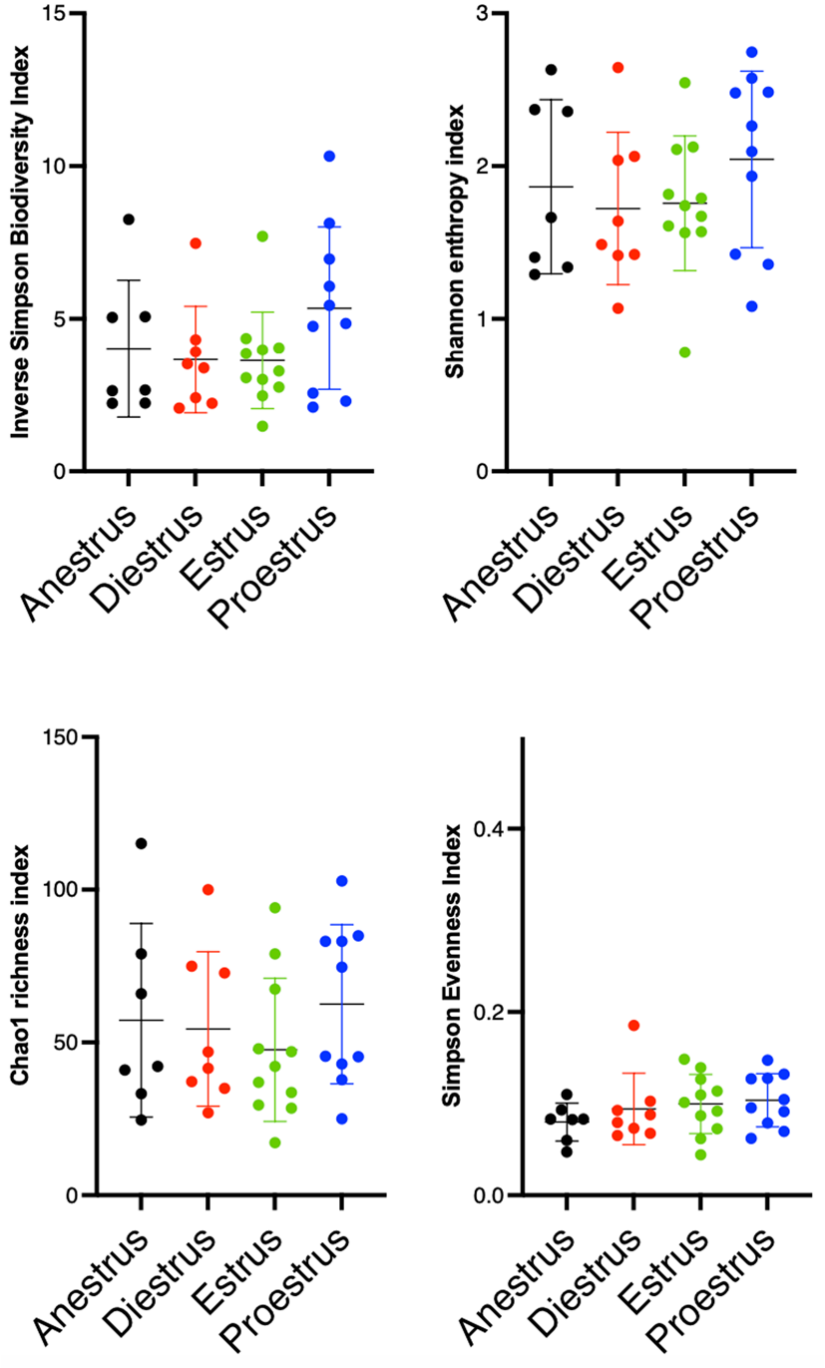
Scatterplots depicting alpha-diversity indices of vaginal microbiota at each phase of the estrous cycle. Each dot represents a subsample. Bacterial intrinsic diversity was calculated using the reciprocal Simpson Biodiversity index, bacterial genus richness was calculated using the Chao1 index and bacterial genus evenness was calculated using the Simpson index. No significant differences were found between the groups based on a Friedman test. Horizontal lines represent the mean, and error bars indicate the 95% CIs for each group and time point

The vaginal bacterial ecosystem was assessed at the genus level. Alpha diversity, richness and evenness of the bacterial populations did not change significantly throughout the different cycle phases (Fig. 3). AMOVA-based cluster analysis showed significant differences between samples from different cycle phases (anestrus, diestrus, estrous and proestrus, *p* < 0.0001). Paired analysis showed significant differences between anestrus and estrous (*p* < 0.0001), anestrus and proestrus (*p* < 0.0001), diestrus and estrous (*p* < 0.0001), diestrus and proestrus (*p* < 0.0001), and estrous and proestrus (*p* < 0.0002). There was no significant difference between anestrus and diestrus. HOMOVA testing was statistically significant (*p* = 0.0035). However, paired analysis did not yield statistically different results.

**Fig. 4:**
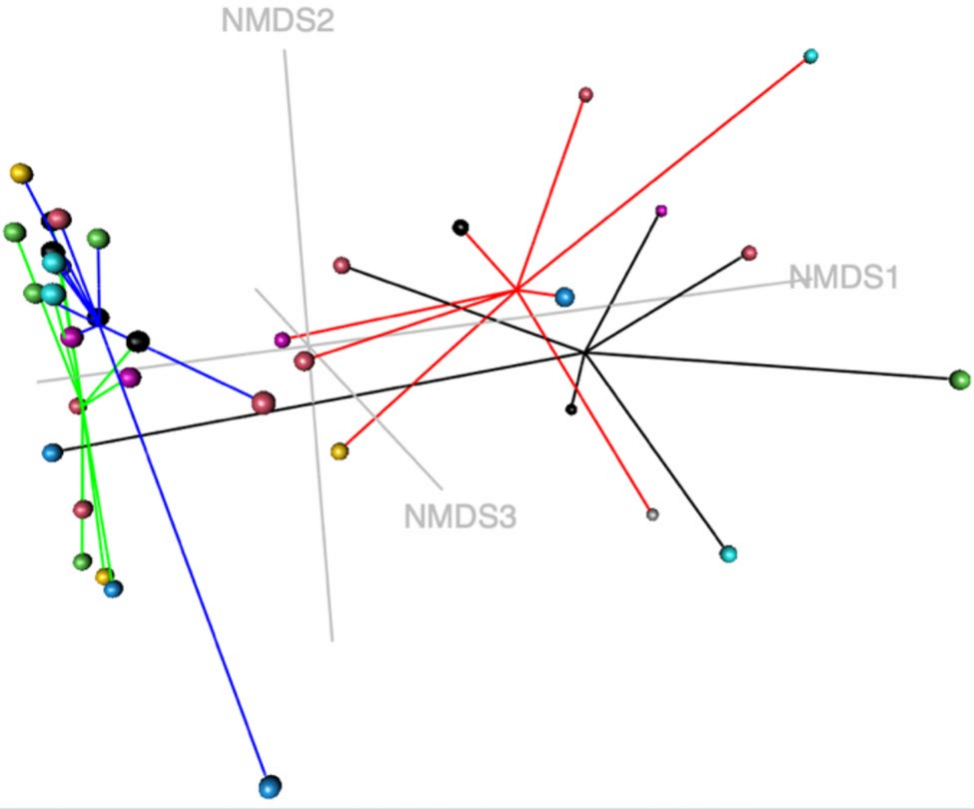
Non-metric multidimensional scaling model (k = 3, stress = 0.0836) based on a Bray-Curtis dissimilarity matrix of the vaginal microbial profiles during estrous cycle phases. Colored dots represent subsamples in the different cycle phases (black: anestrus, red: diestrus, green: estrous, blue: proestrus). AMOVA-based cluster analysis showed significant differences in variance between all estrous cycle phases, except between anestrus and diestrus (p-value = 0.0001). HOMOVA testing showed statistically significant differences (*p* = 0.0035)

The beta-diversity of the vaginal microbial profile was visualized using a non-metric dimensional scaling (NMDS) model based on a Bray-Curtis dissimilarity matrix including samples from the different cycle phases (anestrus, diestrus, estrous and proestrus, Fig. 4).

Differential population abundance in cycle phases was evaluated using a negative binomial Wald test in the DESeq2. The abundance of bacterial populations was not significantly different in anestrus and diestrus samples. In contrast, the abundance of five genera (*Prevotella*, *Variovorax* and *Porphyromonas* and two contaminants—*Rheinheimera* and *Corynebacteriale*) were significantly different in proestrus and estrous samples. Notably, there were significant differences in the abundance of 32 genera, including *Parvimonas, S5.A14, Peptostreptococcae, Anaerovoracacaea genus, Solobacterium, Porphyromonas, Fusobacterium, Alloprevotella, Peptococcus, Fusobacteriales, Porphyromonadacae, Johnsonella* and six contaminants (*Flavobacteriaceae* genus, *Corynebacteriales* genus, *Rheinheimera, Pelomonas, Pseudomonadaceae* genus and *Escherichia-Shigella*), between anestrus and estrous samples. There were also significant differences in the abundance of 28 genera, including *S5.A14a, Parvimonas, Porphyromonadacae, Anaerovoracaceae, Peptostreptococcaceae, Solobacterium, Alloprevotella* and seven contaminants, between anestrus and proestrus samples. Similarly, there were significant differences in the abundance of 27 genera, including *S5.A14a, Porphyromonadacae, Fusobacterium* and 10 contaminants, between diestrus and proestrus samples. There were also significant differences in the abundance of 33 genera, including *S5.A14a, Porphyromonadacae, Fusobacterium, Bacteroides, Peptostreptococcus, Johnsella, Parvimonas, Peptostreptococcaceae, Prophromonadaceae* and 11 contaminants between diestrus and estrous samples. These results are summarized in Table 2.

**Table 2:** Differential bacterial population abundance in estrous cycle phases is represented by the adjusted p-value (padj) with DESEQ2 test values. The higher abundance in the first group compared to the second is represented by a log2fold change positive. The possible contaminants according to the correlation test are in bold. The possible contaminants according to the Decontam test are underlined

Anestrus-Estrous	log2FoldChange	padj	Estrous-Proestrus	log2FoldChange	padj	Anestrus-Proestrus	log2FoldChange	padj	Diestrus-Proestrus	log2FoldChange	padj	Diestrus-Estrus	log2FoldChange	padj
***Flavobacteriaceae*** **genus**	20.50	1.05926E-22	*Prevotella*	31.43	4.38087E-33	***Flavobacteriaceae*** **genus**	23.03	1.84734E-27	***Flavobacteriaceae*** **genus**	28.25	1.11844E-44	***Flavobacteriaceae*** **genus**	25.73	5.90348E-39
*Corynebacteriales* genus	25.36	1.41072E-22	***Rheinheimera***	-16.12	1.49931E-13	***Pelomonas***	22.21	9.15592E-19	***Pelomonas***	27.92	3.43701E-32	***Rheinheimera***	25.88	1.10481E-35
***Rheinheimera***	20.14	6.14841E-20	*Variovorax*	17.21	4.1406E-07	*Prevotella*	21.63	8.26798E-13	*Prevotella*	23.67	2.15437E-16	***Pelomonas***	24.90	4.05461E-27
***Pelomonas***	19.19	6.68813E-15	*Corynebacteriales* genus	-13.67	5.20347E-07	*Rhodococcus*	25.16	2.37102E-12	*Rhodococcus*	24.90	6.33797E-13	*Corynebacteriales* genus	25.69	2.50813E-25
*Rhodococcus*	23.96	8.61807E-12	*Porphyromonas*	5.46	0.001965367	*Variovorax*	21.50	4.94071E-10	*Variovorax*	23.29	1.55098E-12	***Escherichia Shigella***	10.94	3.96533E-14
*Pseudomonadaceae* genus	12.56	1.39975E-09				*Pseudomonadaceae* genus	12.89	1.07674E-09	***Escherichia Shigella***	10.13	1.92812E-12	*Rhodococcus*	23.70	1.42357E-12
*Conchiformibius*	22.19	2.0297E-06				*Corynebacteriales* genus	11.69	6.26809E-05	*Pseudomonadaceae* genus	12.78	3.3036E-10	*Conchiformibius*	27.57	1.16139E-10
*Parvimonas*	-6.42	5.23916E-06				*Conchiformibius*	19.43	7.23703E-05	*Conchiformibius*	24.81	2.62996E-08	*Pseudomonadaceae* genus	12.44	2.63062E-10
*S5 A14a*	-11.32	2.62116E-05				*Abiotrophia*	19.14	7.23703E-05	*Abiotrophia*	23.75	6.16636E-08	*S5 A14a*	-13.89	2.74823E-08
***Escherichia Shigella***	6.55	4.4847E-05				*S5 A14a*	-10.23	0.000292341	*Pseudomonas*	12.23	4.85539E-07	*Porphyromonas*	-7.97	9.7955E-08

Differences in bacterial abundance were evaluated using a negative binomial Wald test in the DESeq2 R package. Differences were considered significant if the corrected p-value < 0.05 (Benjamini-Hochberg False Discovery Rate multi-testing correction). Bacteria were suspected to be a contaminant when the correlation coefficient (r) between the microbiota abundance and presence of the bacteria in the sample was < -0.5. Bacteria suspected to be contaminants are shown in bold and bacteria present at a level lower than 1% of the total population are highlighted in dark grey.

### Urinary microbiota

**Fig. 5:**
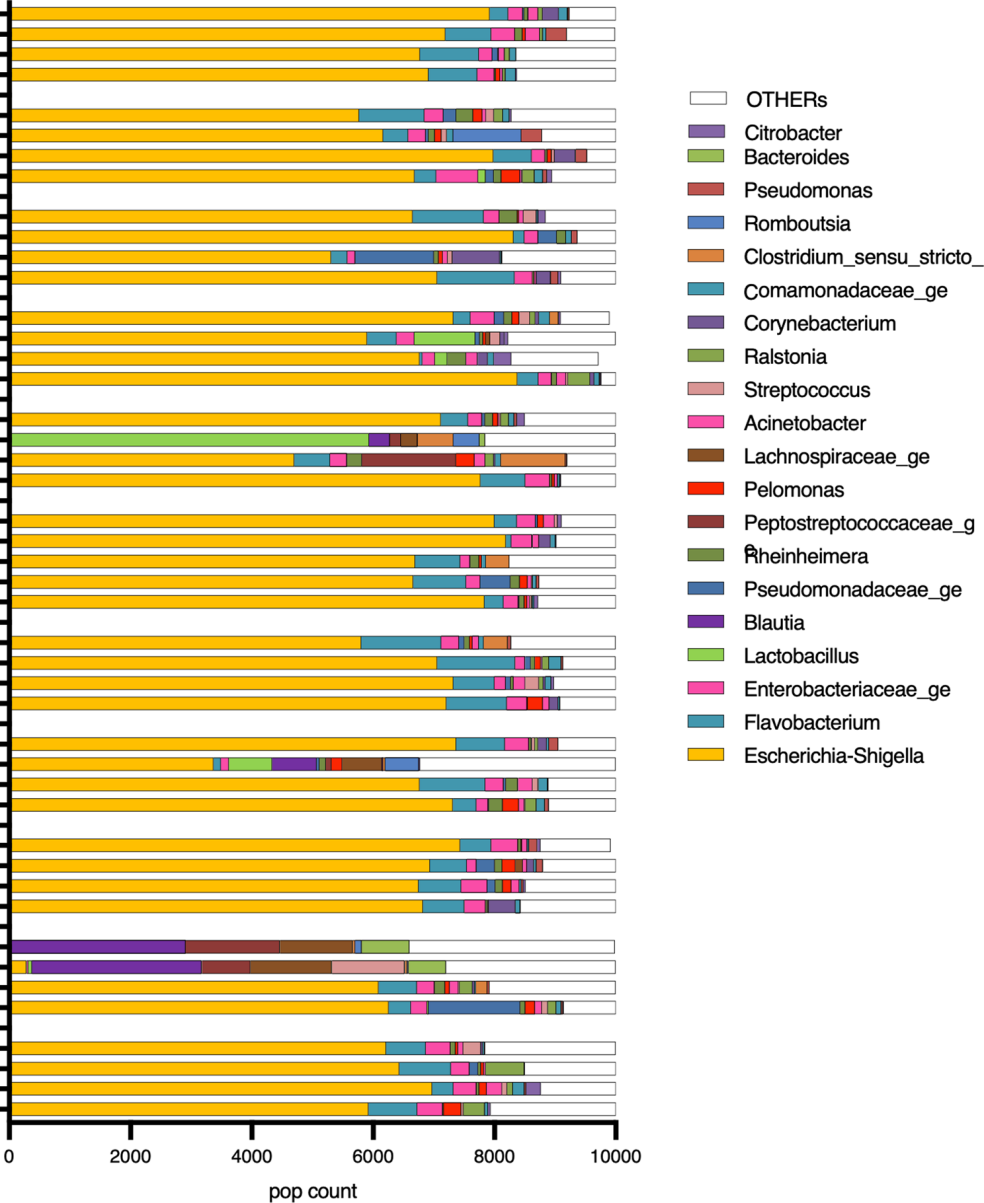
Urinary genus

A total of 351 genera were identified in the urinary samples during the different estrous cycle phases. The most abundant bacterial populations belonged to the genera *Escherichia-Shigella*, *Flavobacterium*, *Enterobacteriaceae* genus, *Pseudomonadaceae* and *Rheinheimera*, which represented 67%, 6.5%, 3%, 1.3% and 1%, respectively, of the total bacterial population (Fig. 5).

**Fig. 6:**
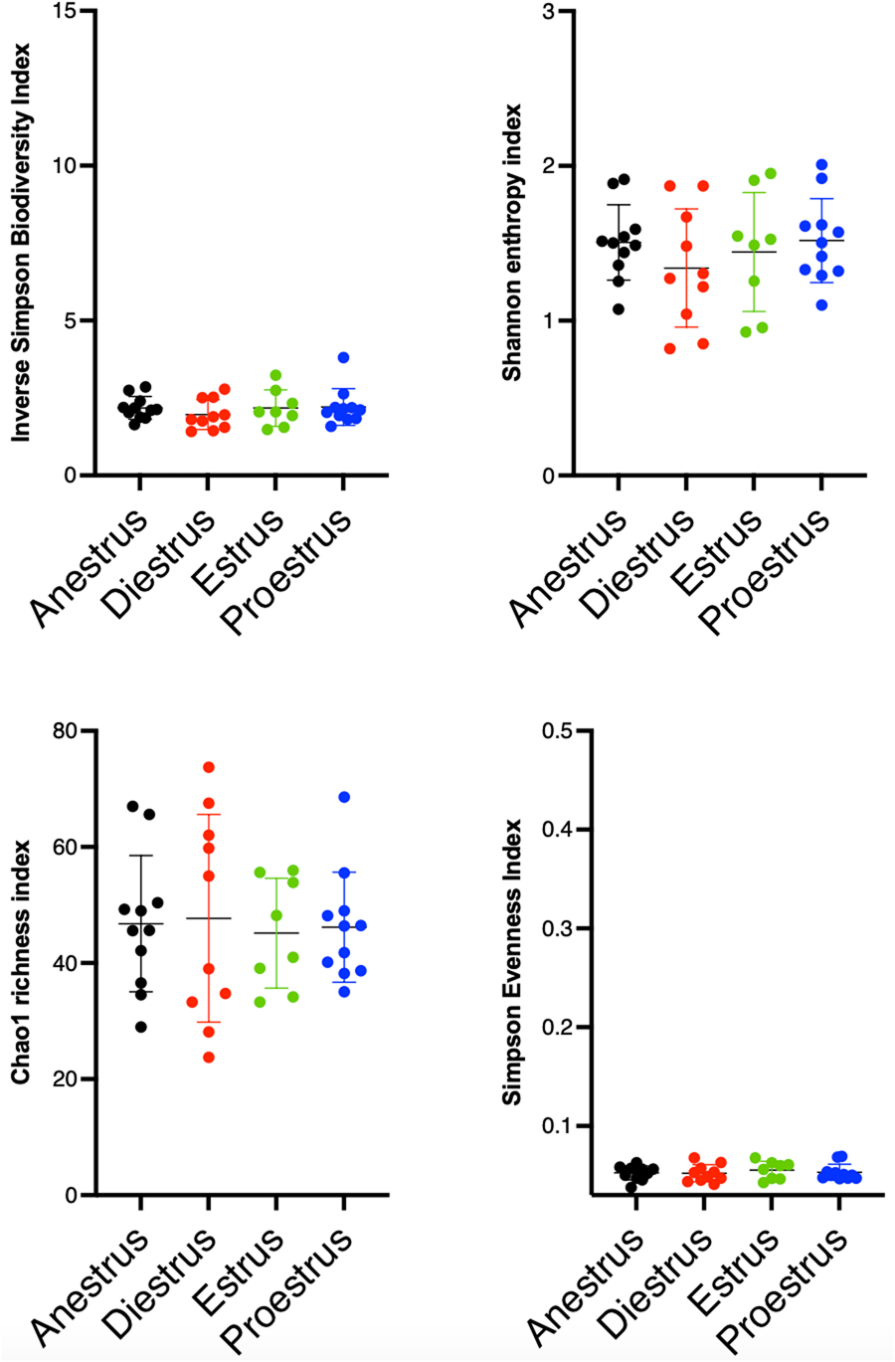
Scatterplots depicting alpha-diversity indices of urinary microbiota at each phase of the estrous cycle. Each dot represents a subsample. Bacterial intrinsic diversity was calculated using the reciprocal Simpson Biodiversity index, bacterial genus richness was calculated using the Chao1 index and bacterial genus evenness was calculated using the Simpson index. No significant differences were found between the groups based on a Friedman test. Horizontal lines represent the mean, and error bars indicate the 95% CIs for each group and time point

The urinary microbial ecosystem was assessed at the genus level. There were no significant differences in alpha diversity, richness and evenness of the bacterial populations throughout the different cycle phases (Fig. 6).

**Fig. 7:**
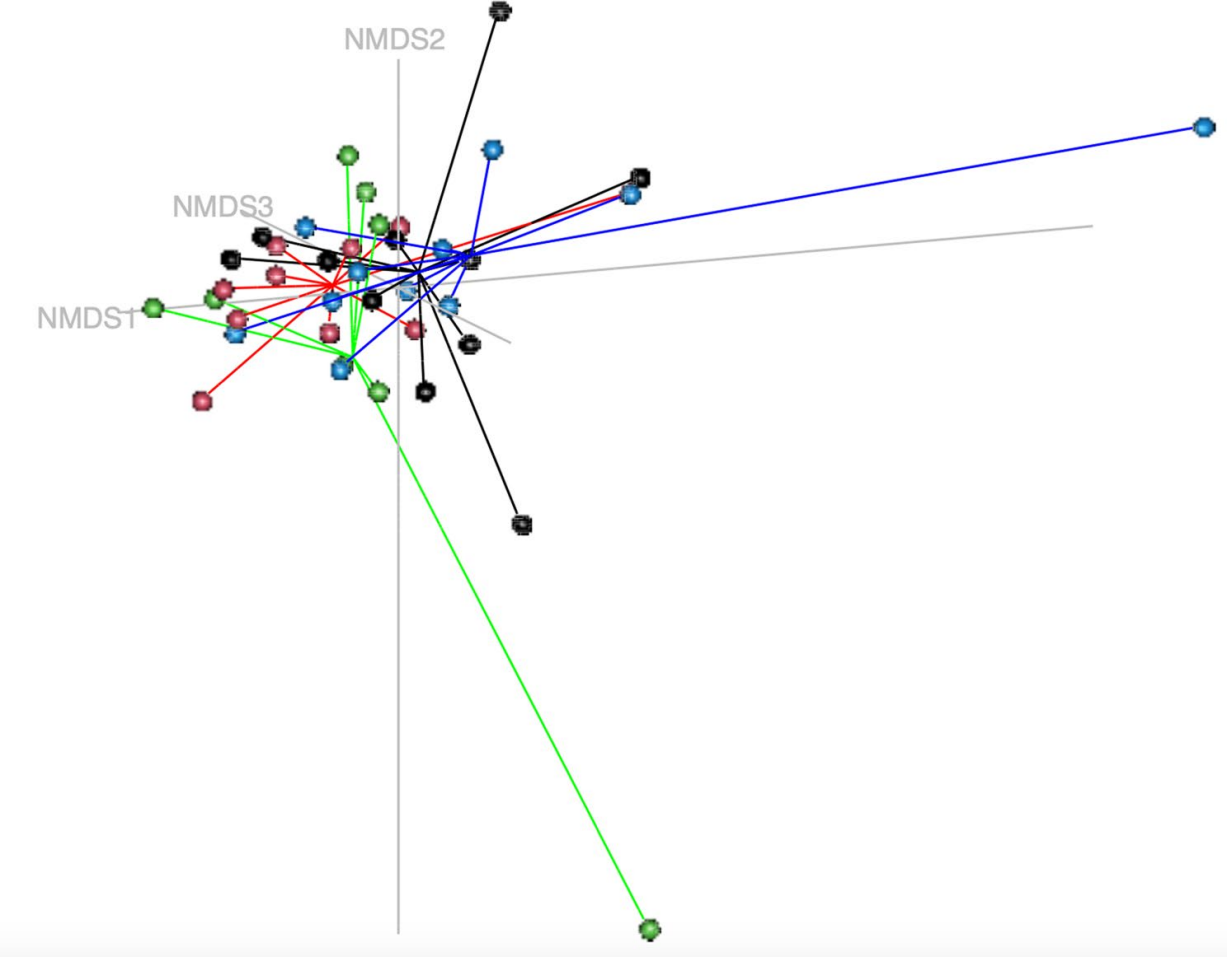
Non-metric multidimensional scaling model (k = 4, stress = 0.084) based on a Bray-Curtis dissimilarity matrix of the urinary microbial profiles during estrous cycle phases. Colored dots represent subsamples in the different cycle phases (black: anestrus, red: diestrus, green: estrous, blue: proestrus). AMOVA- and HOMOVA analysis showed no significant differences between estrous cycle phases

AMOVA-based cluster analysis and HOMOVA analysis found no significant differences between samples from the different cycle phases (anestrus, diestrus, estrous and proestrus, Fig. 7).

Urinary and vaginal microbiota were compared and no statistically significant correlation was observed.

## Discussion

To the authors’ knowledge, this is the first study investigating the changes in both urinary and vaginal microbiomes during the estrous cycle in healthy female dogs. We found significant differences between the most prevalent bacteria present in the vagina and those in the urine. Our data also showed significant changes in the prevalence of various bacterial genera in the vagina during the different phases of the estrous cycle. In contrast, estrous cycle phases did not affect bacterial prevalence in urine samples.

The vaginal microbiome includes bacteria, fungi, viruses, archaea, and candidate phyla radiation bacteria. Within this mix, bacteria are the most prevalent microorganisms in the vagina. Characterization of tissue-specific microbiomes can help identify pathologic microbial changes in various disease states. In this study, we chose to describe the bacterial population in urinary and vaginal samples at the genus level. This allowed precision while avoiding noise associated with identification by species. This best suited the purpose of our research, which was to characterize and compare the urogenital microbiota in the different phases of the estrous cycle in healthy bitches.

Early studies characterizing vaginal microbiota by aerobic and anaerobic culture methods found that *E. coli* and *S. pseudintermedius* were the most common isolates in bitches [6, 27]. These results are likely due to the limitations of routine culture. More recent studies using 16 S rDNA gene sequencing to identify bacterial populations have reported a more diverse vaginal microbiome in female dogs. Burton et al. reported *Hydrotalea, Ralstonia, Mycoplasma, Fusobacterium* and *Streptoccocus* to be the predominant genera in the vagina of female dogs. [6] Rota et al. identified *Mycoplasma, Pasteurellaceae* family and *Salmonella* in healthy bitches of various breeds [28] and Hu et al. found *Fusobacteria, Firmicutes, Proteobacteria, Tenericutes* and *Bacteroidetes* in beagles. [19] The results of our study are partly consistent with data from Burton et al. [6] and Hu et al. [19] In our population, *Fusobacterium, Porphyromas*, *Parvimonas* and *Escherichia-Shigella* were the predominant genera in vaginal samples. However, our correlation data suggest that *Escherichia-Shigella* was a contaminant. The data from our study, and from previous studies, suggest that the vaginal microbiome in dogs is significantly different to that of women; in women, the vaginal microbiota is mostly dominated by *Lactobacillus crispatus, Lactobacillus iners, Lactobacillus gasseri* or *Lactobacillus jensenii* [29]. 

Early studies using aerobic and anaerobic culture characterized urine as sterile [30]. However, a more recent study using 16 S rDNA gene sequencing reported bacteria from the phylum *Pseudomonadota*, *Pseudomonas* spp, *Acinetobacter* spp, *Sphingobium* spp and *Bradyrhizobiaceae* to dominate the urinary microbiota in dogs [7]. The results of our study partly overlap with those of the previous report. In our population, *Escherichia-Shigella*, *Flavobacterium*, *Enterobacteria*, *Pseudomonadaceae* and *Rheinheimera* were dominant. Moreover, *Lactobacillus gasse* was identified in the urine of one dog but was not identified in her vaginal microbiota. *Escherichia-Shigella, Enterobacteriaceae* genus, *Pseudomonadaceae* and *Rheinheimera* all belong to the *Pseudomonadota* phylum. In women, *Lactobacillus, Corynebacterium, Streptococcus, Actinomyces, Gardnerella* and *Bifidobacterium* have been observed in urine [3, 4]. These data suggest that the urinary microbiome could be species-specific, as reported for the intestinal microbiota [31]. However, the difference between different study could be due to bias as contamination and analytic and collection method. Mrofchak et al. 2022 suggested in one study that dominant taxa could be shared between humans and dogs [10]. 

To identify potential contaminants, we performed a correlation test between the presence of each genus and the abundance of the total bacterial population in the vagina. Our results suggest that *Escherichia-Shigella, Comamonadaceae, Pseudomonas, Flavobacteriaceae, Enterobacterales, Flavobacterium, Rheinheimera, Pelomonas, Acinetobacter, Saccharimonadales, Chryseobacterium, Parcubactera, Aeromonas, Burkholderiales* and *Paracoccus* are contaminants. In our study, *Escherichia-Shigella, Flavobacterium*, *Enterobacteriaceae* genus and *Rheinheimera* were the dominant genera in urinary microbiota, and we found no correlation between vaginal and urinary microbiota. However, these bacteria have been found in the vaginal microbiota in previous studies. Furthermore, a previous study in dogs found that the genital microbiome was similar to the urinary microbiota [6]. This discrepancy may be due to cross-contamination of the vaginal and urine samples during collection in the study of Burton et al. 2017. As they did not use a sterile speculum for the vaginal swab like we did. Alternatively, this difference may be explained by the difference in methodology sequencing and taxonomic data base that was used (V4).

Sex hormones contribute to the regulation of the vaginal microbiota in women [11–13, 16, 32]. Alpha and beta diversity of the vaginal microbiome varies across the menstrual cycle of women [32]. Moreover, the vaginal microbiome covaries with estradiol level [32]. However, the influence of progesterone is not well known. The results of our study are partially consistent with the results of the human studies. We found that the vaginal microbiota in female dogs also varies across estrous cycle phases. While alpha diversity, which corresponds to the number of taxonomic groups coexisting in the vagina and their distribution of abundances, was not significantly different across cycle phases, beta diversity was significantly different all comparisons, except between diestrus and anestrus. The fact that we observed more stability during the cycle in dogs than has been observed in humans may be due to the fact that we analyzed the microbiota once per cyclic phase, compared with the human study that analyzed vaginal microbiota daily. Additional larger studies with more frequent sampling may help understand this. Our results may also reflect the species-related differences between dogs and humans.

Similar to the results of human studies, our data didn’t demonstrated that progesterone affect the vaginal microbiota, as beta diversity did not differ between diestrus, when progesterone is highest, and anestrus, when there are no significant levels of circulating sex hormones [33]. Furthermore, the variation of beta diversity during estrogenic phases (proestrus and estrous), compared with diestrus and anestrus, suggests that estrogen influences the vaginal microbiome in dogs, as described in women [16]. 

In women, estrogens stimulate vaginal epithelial cell proliferation, with a mid-cycle peak in intracellular glycogen levels in the vaginal mucosa and a subsequent increase in lactic acid-producing microbes, such as *Lactobacillus*, in the vaginal milieu [34]. While estrogens have been shown to similarly effect vaginal cell proliferation in the dog [35], the mechanisms underlying the changes in the microbiome remain to be explored. Moreover, *Lactobacillus* was not identified in the vaginal microbiota in our study population, which suggests a significantly different vaginal environment between women and dogs. However, our results conflict with an earlier study showing that *Lactobacillus* was present in the vagina of dogs and increased as the dogs aged [19]. The difference in vaginal environment between dogs and women, with a pH of 7 and 4.5 respectively, could explain why the microbiota composition is significantly different in the two species [28]. The presence of blood in the vaginal environment during proestrus may also contribute to the changes in beta-diversity due to the presence of iron or the increased pH [29]. Blood may also influence the vaginal microbiota by providing a substrate for growth and proliferation or flushing out bacteria.

Urodynamic studies have shown that the estrous cycle and sex hormones also affect the urinary system by decreasing urethral pressure during estrous and early diestrus [19, 36]. Estrogens induce an increase in the number of alpha-adrenergic receptors and responsiveness of these receptors to sympathetic stimulation [19]. Estrogens also induce an increase in blood flow to urethral tissues [36], which causes an increase in urethral sphincter tone [37]. In contrast, progesterone potentiates beta-adrenergic activity in the urethra of female dogs, leading to a decrease in urethral smooth muscle tone and, therefore, relaxation [37–40]. We hypothesized that sex hormones may affect the urinary microbiome in female dogs. However, we found no differences in the alpha and beta diversity of the urinary microbiota across the estrous cycle phases. This result may be because, although sex hormones influence urethral function, estrogens and progesterone have not been reported to induce changes in the urothelium, as they do in the vagina.

This study has two main limitations. The first is the possible presence of contaminants in the samples despite the aseptic methods used for collection. The second is the small study population that comprised a homogenous group of dogs in terms of breed, age, housing conditions and diet. Therefore, our study population may not accurately reflect a population of pet dogs. Furthermore, in human medicine, diet has been shown to influence urinary and vaginal microbiota [32, 41]. Our study population was fed a strict diet of adult maintenance or light dry food, which, again, is different to healthy pet dogs, which tend to have a more varied diet, including dry food, wet food and table scraps. Therefore, further studies are need to confirm our data in pet dogs and to explore the effect of diet on the urogenital microbiome of dogs.

## Conclusion

This study strongly suggests that estrogen influences the abundance of bacteria in the vaginal microbiota of healthy adult female dogs and provides data comparing the urinary and vaginal microbiota in healthy bitches.

### Electronic supplementary material

Below is the link to the electronic supplementary material.

Supplementary Material 1

Supplementary Material 2

Supplementary Material 3

## Data Availability

All sequencing librairies have been deposited to the Genbank SRA database under bioproject PRJNA1082225.
