# Supplementary material for: Evaluation of the vaginal and urinary microbiota of healthy cycling bitches
Source: BMC Vet Res. 2024 Jul 15;20:315. doi: 10.1186/s12917-024-04104-w (PMC11247753; doi:10.1186/s12917-024-04104-w)

# -Procédure de mise en culture d'échantillons vétérinaires pour la détermination du microbiote.

## 1. Sujet.

Mise en culture d'échantillons vétérinaires afin d'identifier le microbiote propre à chaque prélèvement, de plus pour chaque type de germe identifié effectuer une quantification visuelle, la répondre en pourcentage des germes retrouvés qu'ils soient aérobies ou anaérobies.

### 1.1. Type de prélèvement concerné.

Échantillons urinaires de chien(ne), (culture aérobie)
Frottis vaginaux de chienne, (culture aérobie et anaérobie).
Liquide prostatique de chien, (culture aérobie et anaérobie).

### 1.2. Code MOLIS de l'analyse.

Urines → URET
Frottis vaginaux → VGET
Liquide prostatique → PROSET

## 2. Domaine et intérêt clinique.

Mise en évidence de la stérilité des urines et des liquides prostatiques, sauf dans le cas d'une infection ou d'une contamination avec l'environnement extérieur, (souillure).

Identification du microbiote des frottis vaginaux avec quantification en pourcentage par type de germe identifié.

## 3. Définition, terminologie.

Microbiote : micro-organismes vivant en accord avec son hôte, au sein du site anatomique concerné.

Liquide prostatique : liquide alcalin secrété par la prostate.

Maldi-tof : spectromètre de masse (Matrix Assisted Laser Desorption Ionisation/Time Of Flight).

## 4. Principe général de la méthode.

4.1. Mise en culture.
4.2. Identification.
4.3. Quantification visuelle.
4.4. Encodage des résultats.
4.5. Interprétation.

## 5. Matériel et appareillage.

### 5.1. Instruments.

VITEK MS® Biomérieux® (Maldi-tof) (méthode par spectrophotométrie de masse).

VITEK® 2 Biomérieux® (méthode par tests biochimiques conventionnels).

Incubateur aérobie à 37°C [Marque NUVE modèle EN120] + Jarre d'anaérobiose

Incubateur $CO_2$ à 37°C [Marque NUVE modèle EC160]

Transferpette S BRAND® (0.1 à 2.5 µl)

Microscope. Exemple [Marque NIKON modèle éclipse 55i]

Appareil pour la coloration de préparation microscopique.

[RAL Stainer Réf. 405000]

## 5.2. Petit matériel.

Frottis et récipient stérile pour prélèvement pré-analytique. *

VITEK® MS-DS Biomérieux® (cible) Réf. 410893

Tips 0,1 – 10µl stérile [marque Nerbe plus Réf. 06-360-2018] ou *

GENbag anaer Biomérieux® (générateur d’anaérobiose) Réf. 96124

Carte d’identification VITEK® 2 Biomérieux®

GN Réf. 21341

GP Réf. 21342

ANC Réf. 21347

NH Réf. 21346

YST Réf. 21343

Oese calibrée de 10 µl pour la mise en culture. *

Tube en plastique d’un diamètre de 12 mm pour VITEK® 2 Biomérieux® *

Lame et lamelle pour examen microscopique. *

* (Marque et Réf. En fonction du fournisseur).

# 6. Réactifs.

## 6.1. Réactifs divers.

VITEK® MS CHCA Biomérieux® (Matrice) Réf. 411071
VITEK® MS FA Biomérieux® (Ac formique) Réf. 411072
Solution saline NaCl 0,45% Biomérieux® Réf. V1204
Indicateur d'anaérobiose Anaer Indicator Biomérieux® Réf. 96118
Kit RAL Stainer Gram-Hücker Réf. ZR360215-0000 RAL diagnostics

## 6.2. Milieux de cultures.

CHROMID CPS Elite agar / columbia ANC + 5 % sang de mouton Biomérieux® Réf. 418229
Columbia ANC agar + 5% de sang de mouton Biomérieux® Réf. 43079
Chocolat agar PolyVitex™ Biomérieux® Réf. 43101
Mac Conkey agar Biomérieux® Réf. 43141
Sabouraud Gentamicine Chloramphénicol 2 agar Biomérieux® Réf. 43651
Schaedler agar + 5% sang de mouton Biomérieux® Réf. 43401
Schaedler Neo. Vanco. Agar + 5% sang de mouton Biomérieux® Réf. 433194

## 6.3. Milieux de cultures pour ré isolement.

Chocolat agar + PolyVitex (PVX) Biomérieux® Réf. 43101
Columbia agar + 5% sang de mouton (COS) Biomérieux® Réf. 43041
Sabouraud Dextrose Agar (SDA) Biomérieux® Réf. 43555

# 7. Échantillon.

## 7.1. Nature.

Cf. 1.1. Type de prélèvement concerné.

## 7.2. Préparation.

Non applicable.

## 7.3. Conservation pré et post-analytique.

### 7.3.1. Pré-analytique, géré par le vétérinaire.

Non applicable.

### 7.3.2. Pré-analytique, géré par le laboratoire.

Dès réception des échantillons par le personnel du laboratoire, exemple nos coursiers, ils sont placés dans un bac frigorifique, arrivés au labo ils seront transmis au poste de déballage VT, ou ils seront encodés et étiquetés avec un code barre unique, de même pour la demande les accompagnants, la demandes sera transmise au secrétariat pour l'encodage des analyses demandées ainsi que la signalétique, pour les échantillons ils sont transmis au technologue traitant les échantillons du jour.

### 7.3.3. Post-analytique, après mise en culture, les prélèvements sont stockés en chambre froide dans un bac identifié avec le non du jour pendant une semaine à une température de 5°C +/- 3°C.

# 8. Mode opératoire.

## 8.1. Mise en culture.

### 8.1.1. Échantillons urinaires.

Ensemencer un milieu CHROMID CPS Elite agar / columbia ANC + 5 % sang de mouton Biomérieux® avec une oese calibrée de 10µl et l'incuber à 36°C +/- 1°C en aérobiose.

### 8.1.2. Frottis vaginaux et liquide prostatiques.

Ensemencer les milieux suivants.

#### 8.1.2.1. Culture aérobie.

Columbia ANC agar + 5% de sang de mouton Biomérieux®
Chocolat agar PolyVitex™ Biomérieux®
À incuber à 36°C +/- 1°C sous 5% de $CO_2$
Mac Conkey agar Biomérieux®
Sabouraud Gentamicine Chloramphénicol 2 agar Biomérieux®
À incuber à 36°C +/- 1°C en aérobiose.

#### 8.1.2.2. Culture anaérobie.

Schaedler agar + 5% sang de mouton Biomérieux®
Schaedler Neo. Vanco. Agar + 5% sang de mouton Biomérieux®
À incuber à 36°C +/- 1°C en anaérobiose avec un indicateur d'anérobiose.

8.2. <u>Lecture des milieux et identification de la flore.</u>

Prélever tous les types de germe aérobie et anaérobie, réaliser une identification avec le VITEK MS® Biomérieux® (méthode par spectrophotométrie de masse). Si besoin réisoler pour obtenir une culture non contaminée. Si toutefois l'identification ne donne rien avec le VITEK MS® Biomérieux® (envisageable avec de très rares souches).

Identifier avec le VITEK® 2 Biomérieux® (méthode par tests biochimiques conventionnels utilisable uniquement pour les germes aérobie).
Attention contrairement à la technique précédente qui donne des résultats avec très peu de germe cette technique nécessite une quantité suffisante de germe pour obtenir une suspension de 0,5 McFarland

Si aucune des deux techniques d'identification ne donne un résultat, répondre « Souche atypique, identification impossible avec la méthode par spectrophotométrie de masse ou la méthode par tests biochimiques conventionnels utilisées dans notre laboratoire.».
Code MOLIS saiisb
(Envisageable avec de très rares souches).

### 8.3. Quantification.

La quantification est réalisée de manière visuelle et cela sur les différents milieux de culture aérobie et anaérobie, Elle est exprimée en %.

Pour les germes dont la quantité est négligeables ils ne sont pas précédés de leurs %.

Lorsqu'il y a des germes sans % il faut décoder avec f5 le code MOLIS gsp dans la zone commentaire germe du dernier germe pour lequel le % est répondu, cela décodera : [NB : Les germes n'étant pas précédés de leurs pourcentages respectifs sont présents en faibles quantités, et par conséquent non-quantifiables.].

## 9. Encodage des résultats.

L'encodage des résultats d'identification des germes se fait de manière automatique, après lecture du VITEK MS® Biomérieux® (Maldi-tof) ou du VITEK® 2 Biomérieux®, par transfert de donnés du serveur MYLA Biomérieux® vers le programme MOLIS.

### 9.1. Définition des codes utilisés dans MOLIS.

a → culture négative

esc → *Escherichia coli*

gsp → Les germes n'étant pas précédés de leurs pourcentages respectifs sont présents en faibles quantités, et par conséquent non-quantifiables.

saiisb → Souche atypique, identification impossible avec la méthode par spectrophotométrie de masse ou la méthode par tests biochimiques conventionnels utilisées dans notre laboratoire.

## 10. Interprétation.

Pour la lecture des cultures urinaires, normalement les échantillons sont stériles sauf si la culture est positive suite à une infection urinaire, ou lors d'une contamination avec l'environnement extérieur.
Pour les liquides prostatiques, normalement ils sont stériles, sauf dans le cas d'une infection ou d'une contamination comme pour les échantillons urinaires.
Pour les frottis vaginaux, c'est totalement différent, car ce site anatomique est colonisé par de nombreux types de germe aérobie et anaérobie, sans pour autant qu'il y ait une infection vaginale.

## 11. Annexe.

Flux Culture Urines vétérinaires détermination microbiote.vsdx

Flux Culture Urines vétérinaires détermination microbiote VT.vsdx

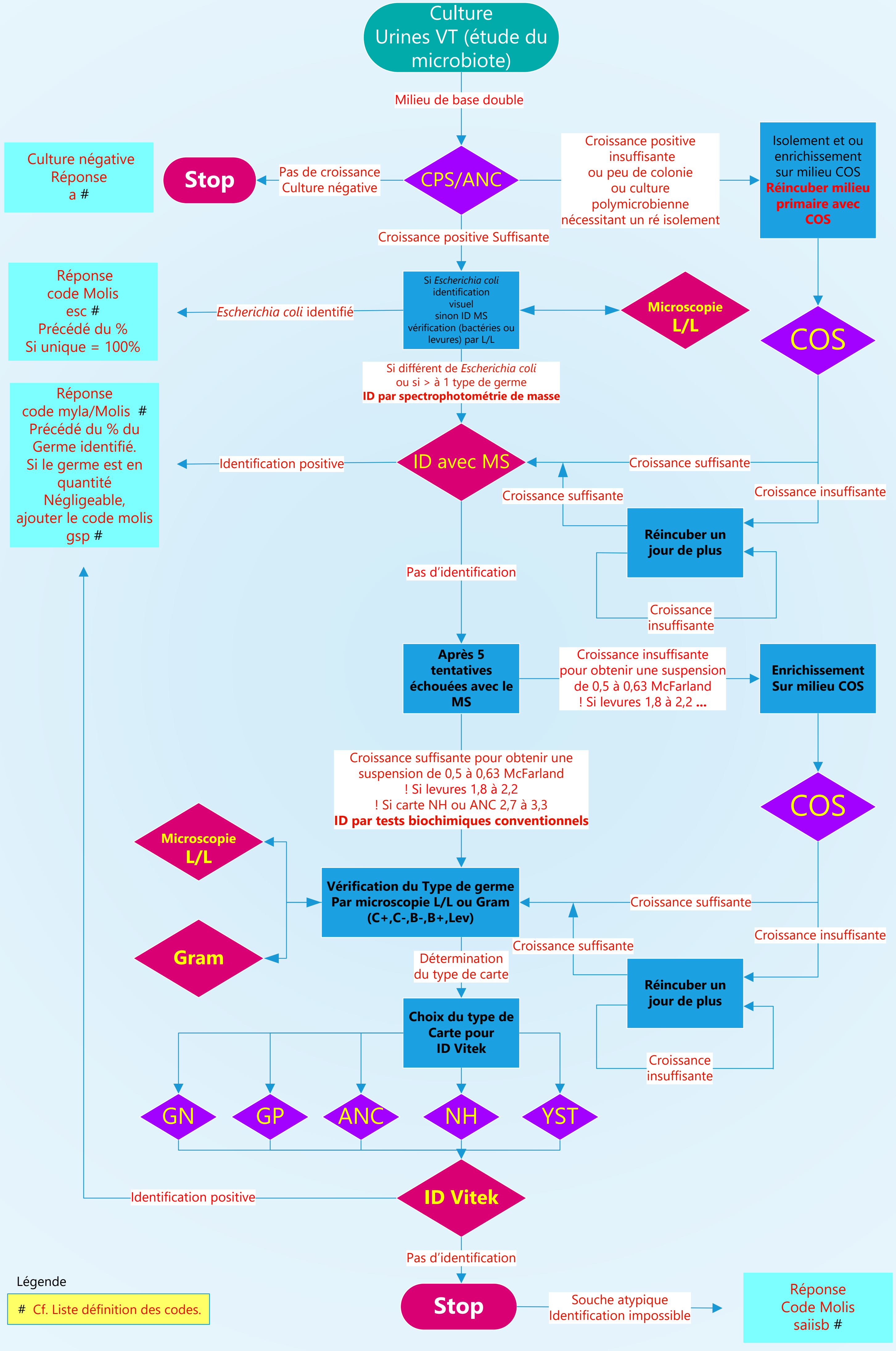

Supplement: Supplementary file 2 — Supplementary Material 2 [file 12917_2024_4104_MOESM2_ESM.pdf]
